# Risk of Death and Heart Failure among Patients with Type 2 Diabetes Treated by Metformin and Nonmetformin Monotherapy: A Real-World Study

**DOI:** 10.1155/2021/5534387

**Published:** 2021-06-10

**Authors:** Siyao He, Xin Qian, Yanyan Chen, Xiaoxia Shen, Bo Zhang, Xiaoping Chen, Xiangjin Xu, Guangwei Li

**Affiliations:** ^1^China Endocrinology and Cardiovascular Disease Centre, Fuwai Hospital, Chinese Academy of Medical Sciences and Peking Union Medical College, Beijing, China; ^2^Department of Endocrinology, China-Japan Friendship Hospital, Beijing, China; ^3^Department of Endocrinology, 900th Hospital, Fuzhou, China

## Abstract

**Background:**

To assess the association of metformin monotherapy with the risk of all-cause deaths and cardiovascular deaths and events in type 2 diabetes patients in real clinical practice.

**Methods:**

This retrospective, observational study comprised patients with type 2 diabetes initially treated with metformin or nonmetformin monotherapy over 2011-2016. Data were extracted from the National Healthcare Big Data database in Fuzhou, China. Propensity score matching (PSM) was performed, matching each patient on metformin to one on nonmetformin in terms of a set of covariates. The primary endpoint was all-cause death, and secondary endpoints were cardiovascular death, heart failure, and heart failure hospitalization. Covariate-adjusted associations of metformin use with all the endpoints were assessed by Cox proportional hazards models.

**Results:**

Among 24,099 patients, 5491 were initially treated with metformin and 18,608 with nonmetformin. PSM yielded 5482 patients in each cohort. During a median follow-up of 2.02 years, we observed 110 and 211 deaths in the metformin and nonmetformin groups, respectively. Metformin was significantly associated with reduced risk of all-cause death (adjusted hazard ratio (aHR) 0.52, 95% confidence interval (CI) 0.39-0.69), cardiovascular death (aHR 0.63, 95% CI 0.43-0.91), and heart failure (aHR 0.61, 95% CI 0.52-0.73), whereas the reduced risk in heart failure hospitalization was not statistically significant (aHR 0.70, 95% CI 0.47-1.02).

**Conclusions:**

In this analysis of electronic health record data from a large database in China, metformin as first-line monotherapy greatly reduced the risk of all-cause death, cardiovascular death, and heart failure in diabetes patients as compared with nonmetformin medications.

## 1. Introduction

Type 2 diabetes mellitus is a progressive metabolic disease characterized by insulin resistance and pancreatic beta-cell dysfunction [1]. Around the globe, more than 415 million people have type 2 diabetes and the number is expected to exceed 693 million by 2045 [1, 2]. China has the largest type 2 diabetes population, and the overall prevalence is nearly 10.9% [3]. Although several antidiabetic agents have been introduced, only 47.7% of the patients in China achieved the target glycated hemoglobin (HbA1c) level of <7% [4]. As diabetes patients are at a higher risk to develop microangiopathy and cardiovascular disease [5], an optimal diabetes management is strongly needed.

The United Kingdom Prospective Diabetes Study (UKPDS) first reported the protective effects of metformin monotherapy against diabetes-related death and death caused by myocardial infarction in overweight patients with type 2 diabetes [6]. Since then, metformin has been recommended as the first-line medication for the management of type 2 diabetes in several guidelines [7–9] and has been used as the preferred initial oral hypoglycemic agent in real practice if lifestyle modification alone fails to maintain adequate glycemic control [10]. However, in China, metformin is less frequently used than sulfonylureas [11], especially in rural areas, because of the low price and high availability of sulfonylureas as well as the rumor that metformin may have severe liver and kidney damage. In addition, the introduction of new oral antidiabetic drugs (OADs) over the past decade has made the decision to start metformin use and overall medication selection even more complicated.

In recent years, an increasing focus has been placed on the effects of OADs on long-term outcomes, especially cardiovascular disease outcomes, rather than solely on short-term hypoglycemic effects. While many new OADs have been demonstrated to have cardiovascular benefits in large-scale randomized controlled trials [12–14], relevant data are still limited for metformin, especially data from real clinical setting. Therefore, in this study, we aimed to assess the association of metformin monotherapy with the risk of all-cause death, cardiovascular death, heart failure, and heart failure hospitalization based on electronic health record data of patients with type 2 diabetes in China.

## 2. Materials and Methods

### 2.1. Participants and Study Design

This is a retrospective, observational study based on regional electronic health record data from the National Healthcare Big Data database in Fuzhou, Fujian Province, China. This database covers more than 23 million patients from 37 secondary and tertiary hospitals with more than 2 billion medical records in Fuzhou from September 2001 to January 2018. Demographics, medical history, laboratory measures, prescription data, and other clinical information generated from healthcare service were recorded in the database. For this analysis, a total of 177,950 type 2 diabetes mellitus patients from 24 general hospitals, diagnosed between January 1, 2011, and December 31, 2016, were evaluated against inclusion/exclusion criteria.

**Figure 1:**
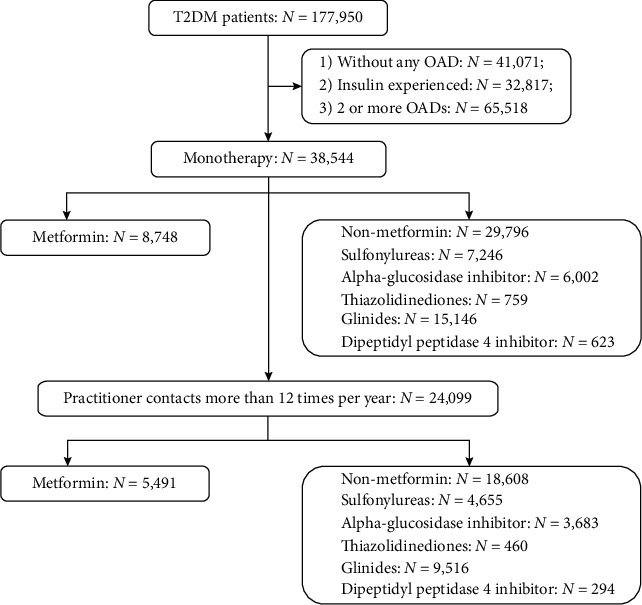
Patient enrollment. OAD = oral antidiabetic drug; T2DM = type 2 diabetes mellitus.

Patients were included in the analysis if (1) they had been diagnosed with type 2 diabetes mellitus (E11 by the International Classification of Diseases, 10^th^ Revision, ICD-10); (2) the first diagnosis of type 2 diabetes mellitus was between January 1, 2011, and December 31, 2016; (3) they had received OAD monotherapy; and (4) they had >12 hospital visits per year for follow-up for diabetes. Patients with a diagnosis of type 1 diabetes, gestational diabetes, and other types of diabetes, not using any OADs (*n* = 41,071), or having been prescribed insulin (*n* = 32,817) or two and more OADs (*n* = 65,518) were excluded. Another 14,445 patients were excluded as they had ≤12 hospital visits per year. Finally, a total of 24,099 patients on OAD monotherapy were included in the analysis and were grouped into metformin monotherapy (*n* = 5491) or nonmetformin monotherapy (*n* = 18,608), which includes sulfonylureas (*n* = 4655), alpha-glucosidase inhibitor (*n* = 3683), thiazolidinediones (*n* = 460), glinides (*n* = 9516), and dipeptidyl peptidase 4 inhibitor (*n* = 294) (Figure 1). The study design was reviewed and approved by the Chinese Ethics Committee of Registering Clinical Trials (ChiECRCT-20190178).

### 2.2. Outcomes and Covariates

Data on demographics, relevant laboratory measures, and comorbidities at the first diagnosis and follow-up visits were extracted. In clinical practice, as per the guidelines of the Chinese Diabetes Society, patients with type 2 diabetes are recommended to have a follow-up visit every 3 months at the beginning of their treatment and then once every 6 months when their HbA1c reaches the target of <7% [15]. In this study, because of the nature of retrospective analysis, we included available data from each follow-up instead of selecting data from fixed follow-up dates.

The primary endpoint was all-cause death, defined as death from any cause from the time of the first prescription to the death date. The secondary endpoints were (1) cardiovascular death, which was defined as deaths that resulted from acute myocardial infarction, heart failure, stroke, and sudden cardiac death [16]; (2) heart failure (I50, I11.000, I13.000, I13.200, and I24.900-901 by ICD-10); and (3) heart failure hospitalization, defined as hospital admission led by a heart failure event [17]. The censor date for all analyses was defined as the earliest end of a patient's recorded data, their date of transfer to an alternative OAD, or their last prescription for monotherapy.

The following demographic and clinical information of type 2 diabetes patients was subjected to adjustment to minimize confounding effects: age, sex, hypertension, hyperlipidemia, coronary heart disease, and history of stroke.

### 2.3. Propensity Score Matching (PSM)

PSM was conducted to match patients on metformin monotherapy with those on nonmetformin therapy, balancing the potential confounders. Propensity score was calculated for each patient by using logistic regression, in which potential confounders, including age, sex, hypertension, hyperlipidemia, coronary heart disease, and stroke history at baseline, were taken as independent variables and treatment group as dependent variable. The nearest neighbor 1 : 1 matching was then implemented, matching each person of the metformin group with a person of the nonmetformin group who has the closest propensity score. A caliper of 0.1 of the standard deviation of the logit of the propensity score was used to define the differences within which matches were considered acceptable (i.e., a match outside of the caliper distance would not be included in the final dataset). To assess the balance achieved by the propensity score, the Student *t*-test for continuous variables and the chi-square test (*χ*^2^) for categorical variables were performed to compare the distribution of potential confounders between the two groups. Imbalanced variables after PSM were taken as covariates in the following multivariable regression analyses.

### 2.4. Statistical Analysis

Descriptive statistics were used to characterize the demographics, laboratory measures, and comorbidities of patients on metformin or nonmetformin monotherapy, before and after PSM. Continuous variables were presented as mean and standard deviation after testing for normality by the Shapiro-Wilk test and were compared using the Student *t*-test. Categorical variables were presented as frequency and percentages and were compared using the *χ*^2^ test. The follow-up person-year was defined as the time interval from the first prescription record to the last record or the censor date in the database. The incidence of primary and secondary outcomes (all-cause death, cardiovascular death, heart failure, and heart failure hospitalization) during follow-up was analyzed using cumulative incidence, calculated as the number of events divided by the corresponding follow-up person-years. Cumulative incidence between groups was compared by the log-rank test.

Multiadjusted Cox proportional hazards models controlling for imbalanced variables after PSM were used to assess the association between use of metformin and the outcomes, and adjusted hazard ratios (aHRs) and 95% confidence intervals (CIs) were calculated. Additional analyses were then conducted by age group, sex, and having or not having hypertension, hyperlipidemia, coronary heart disease, and history of stroke. Statistical significance was set at 0.05 in this study. All statistical analyses were performed using SAS version 9.4 (SAS Institute Inc., Cary, NC, USA).

## 3. Results

Baseline characteristics of patients before and after PSM are shown in Table 1. Prior to PSM, patients on metformin monotherapy were generally younger (56.6 ± 15.8 vs. 60.7 ± 14.6 years, *p* < 0.001) and had a significantly higher proportion of hypertension (67.0% vs. 55.9%, *p* < 0.001), hyperlipidemia (13.2% vs. 9.4%, *p* < 0.001), coronary heart disease (49.2% vs. 45.7%, *p* < 0.001), and cancer (1.9% vs. 1.6%, *p* < 0.001) compared with those treated with nonmetformin. PSM yielded 5482 patients in the metformin and nonmetformin groups each. Except for age and coronary heart disease, individuals from the two groups shared similar demographic characteristics, laboratory measures, and proportion of comorbidities. Approximately 45% of the patients were men, 67% aged <65 years, and the HbA1c level was on average 6.8 ± 1.3% for the metformin group and 7.2 ± 1.4% for the nonmetformin group (*p* = 0.862).

**Figure 2:**
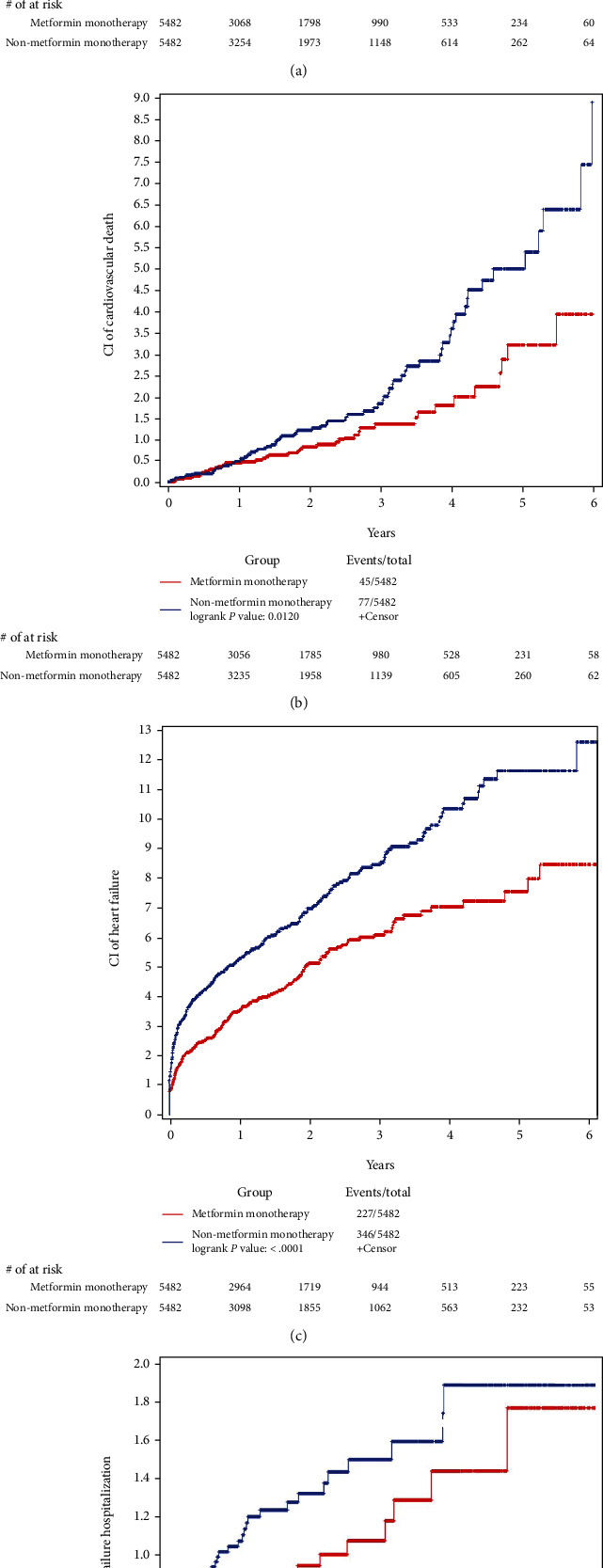
Cumulative incidence among a propensity score-matched cohort of patients on metformin and nonmetformin monotherapy: (a) all-cause death; (b) cardiovascular death; (c) heart failure; (d) heart failure hospitalization. CI = cumulative incidence.

Figure 2 shows the cumulative incidence rates of all-cause death, cardiovascular death, heart failure, and heart failure hospitalization in both the metformin and nonmetformin monotherapy groups. During a median follow-up of 2.02 years (interquartile range (IQR) 1.07-3.36 years), we observed 110 and 211 deaths in the metformin and nonmetformin groups, respectively. The cumulative incidence of all-cause death increased continuously over follow-up and reached 0.87%, 3.38%, and 7.29% at 1-year, 3-year, and 5-year follow-up, respectively, among patients treated with metformin (Table 2). The estimates among those on nonmetformin therapy were significantly higher throughout the entire study period (log-rank *p* < 0.0001) (Figure 2(a)), with a 1-year, 3-year, and 5-year cumulative incidence of 1.85%, 5.13%, and 11.91%, respectively (Table 2). Similar patterns were observed for cardiovascular death (Figure 2(b)), heart failure (Figure 2(c)), and heart failure hospitalization (Figure 2(d)). The 5-year cumulative incidence was 3.19% for cardiovascular death, 7.55% for heart failure, and 1.77% for heart failure hospitalization among patients treated with metformin, as compared to 4.97%, 11.61%, and 1.89%, respectively, for those treated with nonmetformin (Table 2). However, the differences were not statistically significant for heart failure hospitalization (log-rank *p* = 0.0637).

**Figure 3:**
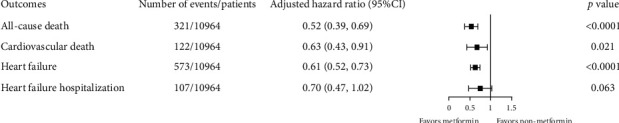
Associations of metformin with all-cause death, cardiovascular death, heart failure, and heart failure hospitalization. Hazard ratios were estimated from multivariable Cox models controlling for age and coronary heart disease (imbalanced variables after PSM). CI = confidence interval.

The associations of metformin with all-cause death, cardiovascular death, heart failure, and heart failure hospitalization estimated by multivariable Cox models controlling for age and coronary heart disease (imbalanced variables after PSM) are shown in Figure 3. Patients treated with metformin were associated with 48% reduced risk of all-cause death than those treated with nonmetformin (aHR 0.52, 95% CI 0.39-0.69). Reduced risk was also observed in the metformin group for cardiovascular death (aHR 0.63, 95% CI 0.43-0.91), heart failure (aHR 0.61, 95% CI 0.52-0.73), and heart failure hospitalization (aHR 0.70, 95% CI 0.47-1.02), though the result for heart failure hospitalization was not statistically significant (*p* = 0.063).

**Figure 4:**
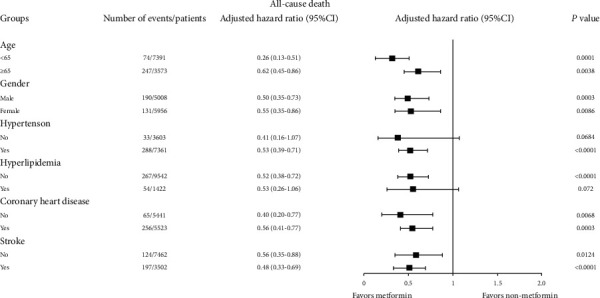
Associations of metformin with all-cause death, by age, gender, and comorbidities. Hazard ratios were estimated from multivariable Cox models controlling for age and coronary heart disease (imbalanced variables after PSM), except for subgroup analysis by coronary heart disease. CI = confidence interval.

In age-specific analyses, much stronger reduction of all-cause mortality was seen in patients aged <65 years (aHR 0.26, 95% CI 0.13-0.51) than in those ≥65 years (aHR 0.62, 95% CI 0.45-0.86) (Figure 4). Men and women experienced similar protective effects of metformin against all-cause death. When stratified by having or not having hypertension, hyperlipidemia, coronary heart disease, or history of stroke, consistent risk reduction of all-cause mortality was observed for patients treated with metformin, though the association among those with hyperlipidemia and those without hypertension was not statistically significant due to a small sample size (Figure 4). In the subgroup analyses for cardiovascular death, heart failure, and heart failure hospitalization (Supplementary Figures S1a–S1c), we also noted generally consistent reduction of risk for metformin across age groups, gender, and having or not having comorbidities, with some estimates not statistically significant.

## 4. Discussion

In this retrospective analysis of electronic health record data from a large database in China, all-cause mortality of diabetes patients was reduced by around 48% among those who accepted metformin monotherapy, as compared with those on nonmetformin monotherapy. Protective effects of metformin were also observed for cardiovascular death and heart failure, whereas the reduced risk in heart failure hospitalization did not reach statistical significance. When stratified by age, gender, and comorbidities, generally consistent results were obtained.

To the best of our knowledge, this is one of the largest studies that evaluated and compared the protective effect of metformin versus nonmetformin towards cardiovascular events and all-cause death in real clinical practice. Our results are in line with and expand the findings of the UKPDS, in which 342 type 2 diabetes patients assigned to receive metformin showed greater reduction in macrovascular and microvascular complications, stroke, and all-cause mortality than those who received other medications (chlorpropamide, glibenclamide, or insulin) for glucose control (total *n* = 951) [6].

Type 2 diabetes has been recognized as a major risk factor for cardiovascular disease [5]. In addition to diabetes patients having a high prevalence of common risk factors for cardiovascular disease, such as obesity and dyslipidemia, studies have found that the increased risks come from some diabetes-specific factors, for example, chronic hyperglycemia, postprandial hyperglycemia, and insulin resistance [18]. Glycemic control has been shown to have a favorable influence on macrovascular complications in the long run [19], which emphasizes the importance of diabetes management in preventing cardiovascular events and deaths among patients. The efficacy of metformin, one of the most used hypoglycemic drugs, in improving glycemic control has long been established [20]. In addition, metformin delivers its protective effects against cardiovascular disease through multiple biochemical pathways, including activation of adenosine monophosphate-activated protein kinase and production of nitric oxide [21]. Our results suggest that metformin could effectively reduce heart failure events, even among those with established cardiovascular disease. A systematic review of 11 studies also found metformin was associated with reduced mortality in patients who have already had heart failure [22].

Over the past decade, the spectrum of hypoglycemic drugs has increased enormously following the development of dipeptidyl peptidase 4 (DPP-4) inhibitors, glucagon-like peptide-1 (GLP-1) receptor agonists, and sodium-glucose cotransporter-2 (SGLT2) inhibitors, allowing individualization of antidiabetic therapy for patients with type 2 diabetes [23]. In recent years, every new antidiabetic therapy was recommended to conduct a cardiovascular outcome trial (CVOT) to evaluate its cardiovascular risk [23]. So far, this has encompassed studies on new hypoglycemic drugs, such as DPP-4 inhibitors, GLP-1 RA, and SGLT2 inhibitors, which have provided a large amount of evidence on the cardiovascular benefits of these drugs, especially among high-risk populations [12–14, 23], and have resulted in the changes of treatment recommendations. Accompanying the rise of new medications was a discussion regarding whether metformin continues to be the first choice for all patients because of limited data on cardiovascular outcomes, even though the safety and efficacy of metformin has been well established in its long-standing history of use. Considering that it might not be practical to conduct large-scale randomized controlled trials for such long-existing medications, observational studies using electronic health data can be a good, if not better, alternative to demonstrate the cardiovascular benefits of metformin. Data obtained from real clinical practice can provide reliable evidence on the protective effects of metformin against cardiovascular events and death in a wider population of varied characteristics across different scenarios, translating the findings of highly controlled trials to externally valid conclusion.

The benefit of metformin use extends beyond cardiovascular disease prevention. A growing body of joint evidence from preclinical research and epidemiological studies has suggested a protective effect of metformin against the development of cancer [24–26], which to some extent explains the observed larger reduction in cumulative incidence of all-cause death than cardiovascular death alone among those treated with metformin. It is also worth noting that in this analysis, we have a higher proportion of women than men and a large proportion of the elderly (aged ≥65 years). Considering that aging is an independent risk factor for multiple chronic conditions [27] and that in the older adults men were more frequently reported to have coronary artery disease and other severe conditions [28], the absolute mortality estimated in this analysis might be somewhat biased. However, since PSM was performed to adjust for age and gender, the estimates of the effects of metformin on the study outcomes shall be accurate, especially when considering that the gender-specific hazard ratios are similar for the primary outcome between men and women.

In addition to a large sample size, the strength of this study includes a good study design and the use of proper, advanced statistical methods. Previous studies were often subjected to immortal time bias; some had not considered time-window bias, and some did not consider inherent time lagging issues when comparing the first-line treatment with second- or third-line treatments [29], whereas in the present study, the data collection was properly designed with time point assessment of metformin therapy, hence the biases for the immortal time lag can be avoided. Additionally, PSM was performed to adjust for a series of confounders so that a more accurate comparison can be obtained.

Some limitations should be noted when interpreting the results. First, because of the nature of retrospective observational study, the baseline characteristics between the metformin and nonmetformin groups were substantially different; however, considering that PSM was performed, the impact on the final results shall be small. Another limitation relates to the limited variables collected in real clinical practice; for example, additional characteristics (such as BMI and body fat), behavioral factors (such as diet, exercise, and smoking), uptake of other medications or therapies (such as cardiac resynchronization therapy and medications for heart failure), and dosage details of OADs were not captured by the electronic health record in a comprehensive manner, which prevented any further assessment and which, otherwise, would be considered to be included in the analysis and might have an impact on the results to some degree. Furthermore, patients with cancer or chronic inflammatory diseases were not excluded from the analysis, which might affect the results, especially the absolute incidence rates, though the impact of cancer on the final results shall be limited because of the balanced proportions between the two groups after PSM (Table 1). Finally, as SGLT2 inhibitors and GLP-1 RA were only introduced in the Chinese market in recent years, data on these medications are limited.

Despite the limitations, this retrospective observational study adds important evidence on the effects of metformin monotherapy in reducing incidence of all-cause death, cardiovascular death, and heart failure. Our results suggest that metformin can be prescribed early in the course of diabetes management to patients, if having no contraindication and if tolerated, to reduce their risks of cardiovascular events and death in the long run. The evidence generated from real clinical practice can provide valuable information to guide clinicians in decision-making.

## Figures and Tables

**Table 1:** Baseline characteristics of patients on metformin and nonmetformin monotherapy before and after propensity score matching.

	Before propensity score matching				After propensity score matching			
	Total (*N* = 24,099)	Metformin monotherapy (*N* = 5491)	Nonmetformin monotherapy (*N* = 18,608)	*p* value	Total (*N* = 10,964)	Metformin monotherapy (*N* = 5482)	Nonmetformin monotherapy (*N* = 5482)	*p* value
Demographics								
Age, in years (mean ± SD)	59.2 ± 15.0	56.6 ± 15.8	60.7 ± 14.6	<0.001	57.9 ± 14.9	56.6 ± 15.8	59.2 ± 13.9	<0.001
Age (% of <65 years)	14,739 (61.2)	3708 (67.5)	11,031 (59.3)	<0.001	7391 (67.4)	3699 (67.5)	3692 (67.4)	0.887
Sex (% of males)	10,475 (43.5)	2497 (45.5)	7978 (42.9)	<0.001	5008 (45.7)	2491 (45.4)	2517 (45.9)	0.618
Hospital level (% of secondary)	5417 (22.5)	1933 (35.2)	3484 (18.7)	<0.001	3846 (35.1)	1924 (35.1)	1922 (35.1)	0.968
Laboratory measurements								
HbA1c level (%) (mean ± SD)	6.9 ± 4.3	6.8 ± 1.3	6.9 ± 5.2	0.115	7.0 ± 1.3	6.8 ± 1.3	7.2 ± 1.4	0.862
HbA1c level group (% of <7%)	1189 (67.2)	385 (65.8)	804 (67.9)	0.391	734 (64.9)	377 (65.5)	360 (64.3)	0.681
Comorbidity								
Hypertension (%)	14,076 (58.4)	3677 (67.0)	10,399 (55.9)	<0.001	7361 (67.1)	3668 (66.9)	3693 (67.4)	0.611
Hyperlipidemia (%)	2483 (10.3)	727 (13.2)	1756 (9.4)	<0.001	1422 (13.0)	720 (13.1)	702 (12.8)	0.609
Coronary heart disease (%)	11,195 (46.5)	2701 (49.2)	8494 (45.7)	<0.001	5523 (50.4)	2694 (49.2)	2826 (51.6)	0.014
Stroke (%)	7574 (31.4)	1763 (32.1)	5811 (31.2)	0.218	3502 (31.9)	1761 (32.1)	1741 (31.8)	0.682
Cancer (%)	398 (1.7)	103 (1.9)	288 (1.6)	<0.001	175 (1.6)	94 (1.7)	86 (1.6)	0.063

Abbreviations: HbA1c: glycated hemoglobin A1c; SD: standard deviation.

**Table 2:** Cumulative incidence rates of all-cause death, cardiovascular death, heart failure, and heart failure hospitalization in the metformin and nonmetformin monotherapy groups after PSM.

Outcomes, % (95% CI)	Metformin monotherapy (*N* = 5482)	Nonmetformin monotherapy (*N* = 5482)
All-cause death		
1-year incidence	0.87 (0.59, 1.16)	1.85 (1.44, 2.27)
3-year incidence	3.38 (2.56, 4.19)	5.13 (4.25, 6.01)
5-year incidence	7.29 (5.34, 9.20)	11.91 (9.79, 13.99)
Cardiovascular death		
1-year incidence	0.43 (0.23, 0.64)	0.47 (0.26, 0.68)
3-year incidence	1.36 (0.84, 1.87)	1.81 (1.26, 2.37)
5-year incidence	3.19 (1.76, 4.60)	4.97 (3.49, 6.43)
Heart failure		
1-year incidence	3.57 (3.02, 4.12)	5.33 (4.68, 5.97)
3-year incidence	6.10 (5.20, 6.98)	8.53 (7.53, 9.52)
5-year incidence	7.55 (6.23, 8.84)	11.61 (9.94, 13.25)
Heart failure hospitalization		
1-year incidence	0.70 (0.45, 0.96)	1.07 (0.77, 1.36)
3-year incidence	1.07 (0.71, 1.43)	1.50 (1.10, 1.90)
5-year incidence	1.77 (0.91, 2.63)	1.89 (1.29, 2.50)

Abbreviations: CI: confidence interval; PSM: propensity score matching.

## Data Availability

The electronic medical record data of the National Healthcare Big Data database used to support the findings of this study may be released upon application to the China Electronics Corporation, who can be contacted at 0086-10-8302 6500.

## Supplementary Materials

Supplementary Materials Figure S1a: associations of metformin with cardiovascular death, by age, gender, and comorbidities. Figure S1b: associations of metformin with heart failure, by age, gender, and comorbidities. Figure S1c: associations of metformin with heart failure hospitalization, by age, gender, and comorbidities.
