# Supplementary material for: Risk of Death and Heart Failure among Patients with Type 2 Diabetes Treated by Metformin and Nonmetformin Monotherapy: A Real-World Study
Source: J Diabetes Res. 2021 Jun 10;2021:5534387. doi: 10.1155/2021/5534387 (PMC8213465; doi:10.1155/2021/5534387)
Supplement: Supplementary Materials — Figure S1a: associations of metformin with cardiovascular death, by age, gender, and comorbidities. Figure S1b: associations of metformin with heart failure, by age, gender, and comorbidities. Figure S1c: associations of metformin with heart failure hospitalization, by age, gender, and comorbidities. [file 5534387.f1.docx]

**
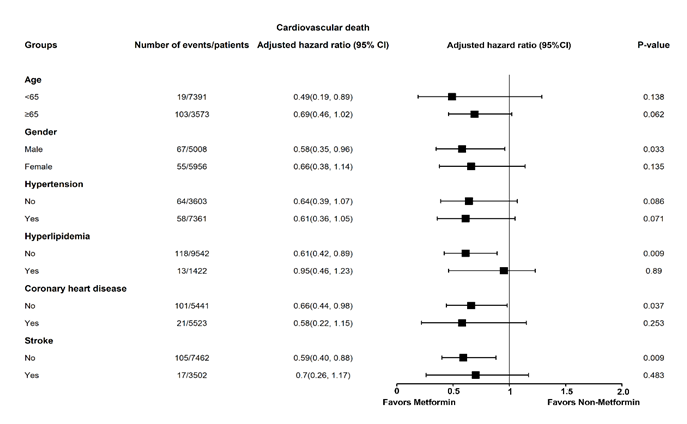
**

**Figure S1a.** Associations of metformin with cardiovascular death, by age, gender, and comorbidities

CI, confidence interval

Hazard ratios were estimated from multivariable Cox models controlling for age and coronary heart disease (imbalanced variables after PSM), expect for sub-group analysis by coronary heart disease.

**
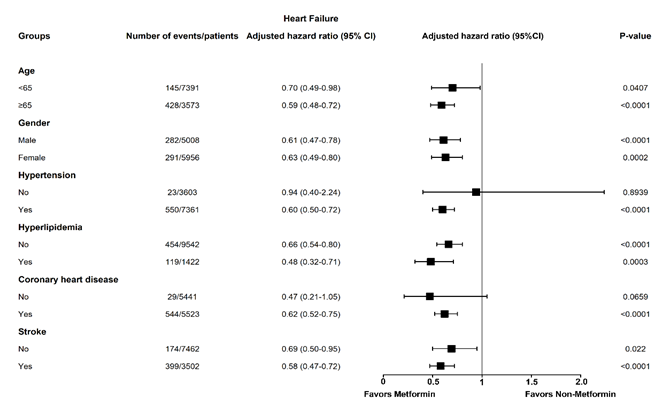
**

**Figure S1b.** Associations of metformin with heart failure, by age, gender, and comorbidities

CI, confidence interval

Hazard ratios were estimated from multivariable Cox models controlling for age and coronary heart disease (imbalanced variables after PSM), expect for sub-group analysis by coronary heart disease.

**
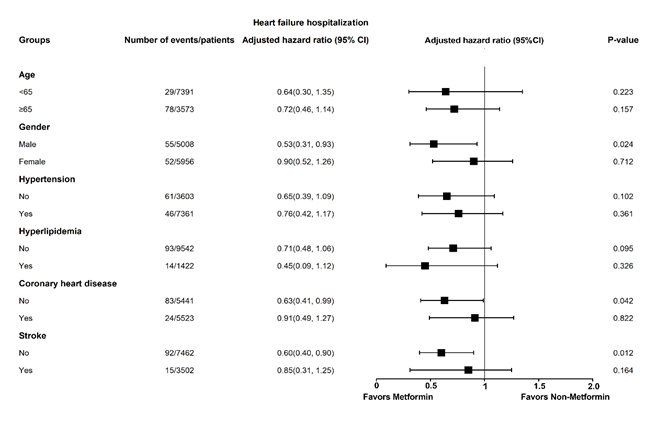
**

**Figure S1c.** Associations of metformin with heart failure hospitalization, by age, gender, and comorbidities

CI, confidence interval

Hazard ratios were estimated from multivariable Cox models controlling for age and coronary heart disease (imbalanced variables after PSM), expect for sub-group analysis by coronary heart disease.
